# The FK506 binding protein 13 kDa (FKBP13) interacts with the C-chain of complement C1q

**DOI:** 10.1186/1471-2210-4-19

**Published:** 2004-09-07

**Authors:** Holger Neye, Eugen J Verspohl

**Affiliations:** 1 KV Niedersachsen, Berliner Allee 22, D-30175 Hannover, Germany; 2 Institute of Medicinal Chemistry, Dept. of Pharmacology, Hittorfstr. 58-62, D-48149 Münster, Germany

## Abstract

**Background:**

The pharmacological action of specific immunosuppressants is mediated by immunophilins. While cyclosporin A binds to cyclophilins, FK506/tacrolimus, rapamycin, and others bind to FK506 binding proteins (FKBPs). Different physiological actions of immunophilins were described but their genuine function, however, remains elusive and is still under investigation. A yeast two-hybrid screen was performed using the FK506 binding protein 13 kDa (FKBP13) as a bait and a fetal liver expression library as a prey.

**Results:**

The C-chain of complement C1q (C1q-C) was detected to interact with FKBP13 in the yeast two-hybrid system and in a protein complementation assay. Neither FKBP12, FKBP25, FKBP52 nor the unrelated immunophilin CypA did react with C1q-C in the yeast system stressing the specificity of the interaction. Binding of C1q-C to FKBP13 could not be prevented in the presence of FK506, demonstrating that possibly other regions than the binding pocket of the drug are responsible for the interaction of the two proteins.

**Conclusion:**

It is concluded that exclusively FKBP13 but no other FKBPs tested so far interact with the C-chain of complement C1q in the two different assays and further work will be initiated to investigate the physiological relevance of the interaction.

## Background

The pharmacological action of cyclosporin A and FK506/tacrolimus is mediated by cytosolic immunophilins namely cyclophilin A and the FK506 binding protein 12 kDa (FKBP12). The drug immunophilin complex binds to and inhibits the protein phosphatase calcineurin thus preventing signal transduction in activated T cells [1]. FKBPs are a family of proteins that were found from procaryotes to humans. At least 10 human FKBPs are known and can be found in every tissue so far analyzed. Some FKBPs bind to and "stabilize" intracellular receptors. For example, FKBP12 and FKBP12.6 interact with the ryanodine Ca^2+ ^channels RyR1 and RyR2, respectively [2,3]. FKBP52 is part of the steroid receptor complex [4]. Another FKBP, FKBP13, is localized in the endoplasmic reticulum. FKBP13 mRNA is up-regulated in the presence of unfolded proteins, e.g. after tunicamycin treatment and heat shock and it is regarded to act as a chaperone [5]. On the other hand FKBP13 was shown to interact specifically with single proteins like a homologue of the erythrocyte membrane cytoskeletal protein 4.1 and a FKBP associated protein 48 kDa [6,7]. Finally, FKBP25 was described to be a nuclear protein and the transcription factor YY1 or histone deacetylases (HDAC1 and HDAC2) specifically interact with this FKBP family member [8,9].

The phylogenetically ancient complement system composed of more than 30 proteins is part of the immunogenic system including a cascade of interacting proteins called C1 to C9. Activation finally leads to lysis of marked cells. C1, the first protein in this cascade, is composed of one C1q and two C1r and C1s proteins. While C1q binds the Fc regions of two IgGs or one IgM molecule, C1r and C1s are known to activate other components of the pathway, namely C4 and C2.

C1q contains 18 polypeptide chains (six A-, six B-, and six C-chains) and is composed of six globular heads linked via six collagen like stalks to a fibril-like central region [10]. Each of the globular heads is formed by association of the C-terminal region of an A-, B-, and C-chain.

In addition C1q seems to play an important role as an immunmodulator in its own and has pathophysiological impact. C1q deficient individuals develop a systemic lupus erythematodes (SLE)-like disease [11] and C1q deficient mice show elevated auto-antibody titres and develop glomerulonephritis and renal damage probably because of accumulation of apoptotic bodies [12]. As C1q can bind to a variety of pathologically relevant targets in an antibody-independent manner it might directly activate cellular functions. Indeed, several receptors for C1q have been described. C1qRp is a putative receptor for phagocytosis enhancement by monocytes [13]. The rodent homologue of C1qRp is tightly regulated during development [14]. C1q was shown to bind to cell lines expressing the complement receptor 1 (CR1) in a saturable manner [15]. A receptor for the collagenous domains of C1q has been purified and was shown to be idenical to calreticulin [16]. Finally, a binding protein for the globular head of C1q (gC1qbp) was isolated [17].

In this work interaction of the C-chain of complement C1q (C1q-C) with a member of the FK506 binding protein (FKBP) family was detected and the specificity of the interaction was further investigated.

## Results

### Yeast two-hybrid screen

The physiological function of immunophilins in endocrine systems is rarely characterized. A commercially available fetal human liver cDNA expression library was used to identify proteins that interact with the immunophilin FKBP13 which is localized in the endoplasmic reticulum. Plasmids encoding FKBP13 without signal peptide fused to the LexA-DBD (pBTM-FKBP13w/oS) and the liver cDNA library were sequentially transformed into yeast L40. Ten million yeast double transformants (corresponding to 3.5 × 10^6 ^independent clones) were screened and selected for histidine prototrophy. Among 216 colonies isolated as His^+^, 21 were found to display beta-galactosidase (β-gal) activity. Plasmids were isolated from each of these clones and were used for retransformation of yeast L40 pretransformed with pBTM-FKBP13w/oS. Six clones remained positive (clone A3, B10, B31, B55, C8, C30) and turned out to code for five different cDNAs after sequence analysis. One of the clones (A3) representing the cDNA of the C-chain of complement C1 corresponding to amino acids 122 to 217 was selected for the present study. The interaction of a second clone with FKBPs was described elsewhere [18].

### Interaction of full length C-chain of C1q with FKBP13 and with other FKBPs

FKBPs share a common binding motif for the immunosuppressant FK506. To check the specificity of the FKBP13/C1q-C interaction, yeast L40 was cotransformed with a plasmid coding for the full length C1q-C-Gal4AD hybrid protein lacking a 26 amino acids signal peptide (pGAD-C1q-Cw/oS) and different plasmids each of them coding for FKBP12, FKBP13w/oS, FKBP25, and FKBP52 fused in frame to the DBD of LexA (pBTM-FKBP12, 13w/oS, 25, and 52). Cotransformants were checked for histidine prototrophy and β-gal activity. Only FKBP13 interacted with the C1q-Cw/oS protein in the yeast two-hybrid system whereas FKBP12, 25, and 52 failed in this respect.

### Interaction of C1q-C with cyclophilin A

Cyclophilins are the second family of immunophilins. To check for cross reactivity, binding of C1q-Cw/oS to the prototype of cyclophilins, cyclophilin A (CypA), was investigated in the same system. Yeast L40 was cotransformed with a plasmid expressing LexA-CypA hybrids (pBTM-CypA) and pGAD-C1q-Cw/oS and was tested for β-gal activity and histidine prototrophy. C1q-Cw/oS and CypA failed to interact in the yeast two-hybrid system.

### Interaction of C1q-C with FKBP13 in a protein complementation assay

**Figure 1:**
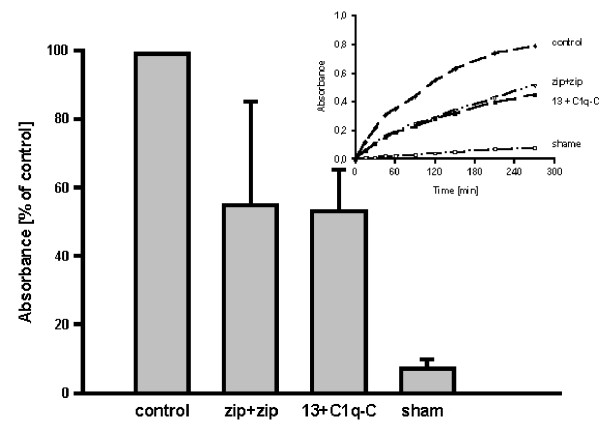
**Interaction of C1q-C and FKBP13 in the protein complementation assay. **INS-1 cells were transfected with pCMVSPORT-Bla[1]M182TlinkBla[2] (control), or were cotransfected with pCDNA3.1Zeo+/F [I]M182T-15-Zip and pCDNA3.1Zeo+/Zip-15-F[2] (Zip+Zip), or were cotransfected with pCMVSPORT-Bla[1]M182TlinkC1q-Cw/oS and with pCDNA-FKBP13linkBla[2] (13+C1q-C). As a negative control cells were transfected with pCDNA-FKBP13linkBla[2] (sham). After lysis of the cells, β-lactamase activity was measured by hydrolysis rates of nitrocefin. The insert resembles the time dependent effect. The absorbances at 490 nm after 90 min of three independent experiments (mean ± S.E.M.) are shown. The control was normalized to 100 %.

In order to verify the results obtained with the yeast two-hybrid screen, the GST pull-down approach was used first. As the expression of C1q-Cw/oS either in a coupled reticulocyte lysate system or in bacteria failed, the interaction of C1q-Cw/oS with FKBP13w/oS was analyzed in a protein complementation assay. INS-1 cells were cotransformed with a plasmid coding for FKBP13w/oS fused in frame via a 15 amino acid peptide linker to aa 198 to 287 of β-lactamase (pCDNA-FKBP13w/oS-Bla2) and a plasmid coding for β-lactamase aa 21 to 196 fused in frame via a 15 amino acid peptide linker to C1q-Cw/oS (pCMVSPORT-Bla[1]M182TlinkCENP-A). As shown in Fig. 1, cotransformation of the hybrid-proteins resulted in β-lactamase activity; the interaction of C1q-Cw/oS with FKBP13w/oS was confirmed by using this second assay.

### Interaction of C1q-C with FKBP13 in the presence of FK506

**Figure 2:**
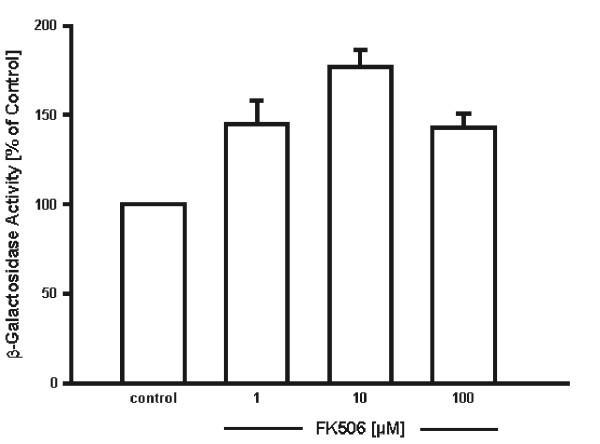
**Influence of FK506 on the interaction of C1q-C with FKBP13. **Yeast L40 was cotransformed with pBTM-FKBP13w/oS and with pGAD-C1q-Cw/oS. Cotransformants were incubated in the presence or absence of FK506 and analyzed as described in material and methods. Shown are the results of 4–8 independent experiments ± S.E.M.. The control (absence of FK506) was defined as 100 %; the solvent ethanol was without influence.

The crystal structure of FK506/FKBP complexes predicts that the recognition site in the endogenous ligand(s) equivalent to FK506 would best be emulated by a Iso-Pro or Leu-Pro motif [19]. FK506 might mimic the binding motif of C1q-C to FKBP13. For this reason, the influence of the drug on the interaction of C1q-Cw/oS with FKBP13w/oS was investigated in the yeast two-hybrid system. Cotransformants of yeast L40 with pGAD-C1q-Cw/oS and pBTM-FKBP13w/oS were incubated in the presence or absence of FK506. Cells were collected and measured for β-gal activity. As shown in figure 2, FK506 does not decrease the interaction of C1q-C with FKBP13, but rather seems to increase the affinity of C1q-C to the FKBP at low micromolar concentrations. The interaction of C1q-C with FKBP13 could not be suppressed, even in the presence of 100 μM FK506.

## Discussion

### The C-chain of complement C1q interacts with FKBP13

A yeast two-hybrid screen was carried out using the FK506 binding protein 13 kDa (FKBP13) as a bait and a human liver expression library as a prey. The screen revealed the C-chain of complement C1q (C1q-C) to interact with FKBP13. Three other FKBPs, namely FKBP12, FKBP25 and FKBP52 failed to interfere with C1q-C under same conditions indicating specificity for the positive result in the yeast system. The interaction of C1q-C with FKBP13 was proved by using additionally a protein complementation assay (PCA), demonstrating that the interaction of C1q-C with the FKBP13 was not an artifact in the yeast two-hybrid system. The interaction of C1q-C with FKBP13 in the PCA seems to be comparable to the interaction of the two GCN4 leucine zippers (Zip) which were used as a positive control. The intensity of the interaction might depend on the efficiency of the cell transfection, cell density, cell growth, and other factors but hydrolysis rates of nitrocefin as a marker of the interaction are clearly different from the negative control which can be best seen after 270 min of incubation (Fig. 1, insert). The verification of the interaction in a classical GST pull-down assay was used elsewhere [18] but failed in this work as the rather small C1q-C protein could not be produced and labelled in vitro (data not shown). Immunoprecipitation of the interacting proteins was not planned since a C1q-C antibody is not available. The interaction, therefore, of C1q-C with different FKBPs was demonstrated in two independent approaches albeit the presence of an additional cellular factor necessary for the interaction can not be excluded with these cellular assays.

### The C-chain of complement C1q does not interact with cyclophilin A

Immunophilins belong to the class of peptidyl prolyl isomerases (E.C. 5.2.1.8). They can be divided into FKBPs that bind for example FK506/tacrolimus or rapamycin and cyclophilins that mediate the pharmacological action of cyclosporin A (CsA). Cyclophilin A (CypA) can be regarded as the prototype of the cyclophilins. Unlike the aforementioned FKBP13, the immunophilin CypA does not interfere with C1q-C in the yeast two-hybrid system underlining the specificity of the interaction of C1q-C with FKBP13.

### FK506 fails to negatively influence the FKBP13 C1q-C interaction

The immunosuppressive drug FK506 is not able to decrease the affinity of C1q-C to FKBP13 in the yeast two-hybrid system even when used in a concentration of 100 micromolar. The common binding motif of FKBPs for FK506 is, therefore, less likely to be uniquely involved in the C1q-C FKBP13 interaction. On the other hand, at low micromolar concentrations FK506 seems to „strengthen" the interaction of C1q-C with FKBP13. Possibly, C1q-C, when produced artificially in yeast, binds to yeast FKBP as well and has to be liberated from these interfering positions by FK506, then contributing to the interaction with the FKBP13-AD hybrid protein. An excess of FK506 should further diminish the C1q-C FKBP13 interaction but the drug cannot be tested in the milimolar range because of its toxicity. The influence of FK506 on the interaction of the two proteins was not investigated in a mammalian cell line because the influence in the yeast system is more likely comparable to other investigations [e.g. [7,18]]. Additionally the immunosuppressive agent is expected to be far toxic in the micromolar range when used in a mammalian cell system. Finally, a negative effect of the immunosuppressive drug on the C1q-C FKBP13 complex could not be demonstrated albeit the involvement of the FK506 binding pocket in the interaction cannot be excluded.

## Conclusions

Using two different experimental approaches, the C-chain of complement C1q was shown to interact with FKBP13. FKBP13, initially used as the bait for the yeast two-hybrid screen in this work, is regarded as an endoplasmic reticulum (ER) protein. It might act as a chaperone but specifically interacts with other proteins like a homologue of the erythrocyte membrane cytoskleletal protein 4.1 or the FKBP associated protein 48 (FAP48), too. FKBP13 was detected in membrane preparations from erythrocytes as well, demonstrating that its expression and maintenance is not restricted to the ER [6]. The C-chain of complement C1q is part of the C1q molecule that is composed of six A-, six B-, and six C-chains, respectively. Interaction of FKBP13 with the A- or B-chain or with the entire C1q molecule has not been investigated yet. The carboxy-terminal part of the C-chain is sufficient to interact with FKBP13 in the yeast two-hybrid system. If the entire C1q molecule interacts with FKBP13, interaction of its globular head with the immunophilin will be likely therefore, as the globular head is is formed by association of the C-terminal regions of an A-, B-, and C-chain. On the other hand, FKBP13 might act as a specific chaperone, triggering C1q-C in the endoplasmic reticulum and preventing the complement protein to interact with other intracellular proteins. Further work will be necessary to elucidate the physiological significance of the interaction of the two proteins and to investigate whether FKBP13 plays an important role in the complement system, too.

## Methods

### Expression vectors

The cDNA encoding FKBP13 amino acids 16–142 was amplified from pBluescript-FKBP13 (a gift from Dr. S. Burakoff, Dana-Faber Cancer Institute, Boston, MA) using specific primers CGCCGGAATTCATGCTGAGCGCCGTG and CGGCTGGATCCGAACAGTCTGGTC. The cDNA encoding full-length FKBP25 was amplified from pBluescript-FKBP25 (a gift from Dr. S. Burakoff, Dana-Faber Cancer Institute, Boston, MA) using the specific primers CGCGCGAATTCAACA GTCTGGTCCCTGATG and GGCGTAGGATCCGGGGTTGACTCCGGGGGC. The cDNA encoding full-length cDNA of CypA was amplified from I.M.A.G.E clone 5264185 (ResGen, Huntsville, AL) by using the specific primers GGTCCGGAATTC ATGGTCAACCCCACCGTGTTC and GGCAGCTGGATCCACAAGTCA AAC TTATTCGAG. After restriction digest, cDNAs were fused to the DNA binding domain of LexA by inserting the EcoRI/BamHI fragments into pBTM116 [20] to give pBTM-FKBP13w/oS, pBTM-FKBP25, and pBTM-CypA, respectively. pBTM-FKBP12 and pBTM-FKBP52 (a gift from Dr. B. Chambraud, INSERM U488, Bicêtre, France) are described elsewhere [7]. Full lenght cDNA of human C1q-C lacking a 26 amino acids signal peptide was amplified from I.M.A.G.E clone AL568589 (ResGen, Huntsville, AL) using the specific primer CACGGAATTCAA GCCAACACAGGCTGCTAC and primer M13 reverse. After restriction digest the cDNA fragment was fused in frame to the GAL4AD by inserting the EcoRI/NotI fragment into pGAD1318 to give pGAD-C1q-Cw/oS. The two vectors pCDNA3.1Zeo+/F [I]M182T-15-Zip and pCDNA3.1Zeo+/Zip-15-F[2], a gift from Dr. S.W. Michnick, Montreal, were used as a positive control in the protein complementation assay and are described elsewhere [21]. The cDNA encoding the TEM-1 β-lactamase gene amino acids 21 to 196 (Bla[1]M182T) was amplified from pGEM11Zf(+) using the primer pairs (i) TTGGGCACCATGGACCCAGAAACG CTGGTGAAAG and GTTAATAGTTTGCGCAACGTTGTTGCCATTGCTACA GGCGACGAGGTGTC and (ii) GCGGCGAAGCTTCAATTGGGCACCATGGAC CCAGAAACG and ACCACCGGATCCGCCAGTTAATAGTTTGCGCAACG. Note that a point mutation leading to M182T was inserted thatway [22]. The cDNA encoding the TEM-1 β-lactamase gene amino acids 198 to 287 and an amino-terminal 15 amino acid linker (linkBla[2]) was amplified from pGEM11Zf(+) sequentially using the primer pairs (i) GGTGGTGGTAGTCGAATTCTACTTACTCTAGCT TCCCGGC and CCAGCTCTCGAGTTACCAATGCTTAATCAGTGAGGCACC (primerBla2XhoI) and (ii) CTGGCGGATCCGGTGGTGGTGGTAGTGGTGGTG GTGGTAGTGGTGGTGGTGGTAGTCGAATTCTAC and primer Bla2XhoI. After restriction digest cDNA of FKBP13 (from pBTMFKBP13w/oS) was fused into the EcoRI/BamHI site of pCDNA3.1 (Invitrogen, Groningen, The Netherlands) and the linkBla[2] fragment was fused into the BamHI/XhoI site of this plasmid to give pCDNA-FKBP13linkBla[2]. After restriction digest Bla[1]M182T and linkBla[2] fragments were sequentially fused into the MunI/BamHI and BamHI/XhoI site of pCMVSPORT6 (Invitrogen, Groningen, The Netherlands) to give pCMVSPORT-Bla[1]M182TlinkBla[2]. After restriction digest cDNA of C1q-C lacking the signal peptide (from pGAD-C1q-Cw/oS) was inserted into the EcoRI/XhoI site of pCMVSPORT-Bla[1]M182TlinkBla[2] to give pCMVSPORT-Bla[1]M182TlinkC1q-Cw/oS. All inserts were verified by sequencing.

### Two-hybrid screen

The human fetal liver MATCHMAKER cDNA library (Clontech, Palo Alto, CA) was a gift from Dr. C. Sorg (Institute of Experimental Dermatology, Münster, Germany). The yeast reporter strain L40, containing two reporter genes, *HIS3 *and *LacZ*, was sequentially transformed with pBTM-FKBP13w/oS and the cDNA library using the lithium acetate method [23]. Double transformants were plated on Minimal SD Base containing the -Leu/-Trp/-His DO supplement (Clontech, Palo Alto, CA). The plates were incubated at 30°C for 5 days. His^+ ^colonies were patched and assayed for β-gal activity. Positive clones, inserted into the pACT2 vector, were rescued and tested for specificity by retransformation into yeast L40 with pBTM-FKBP13w/oS. Different clones, that expressed detectable β-gal activity within four hours, were used for further analysis. All assays of β-gal activity and histidine prototrophy were performed according to standard procedures [24].

### Protein complementation assay

The protein complementation assay (PCA) was performed as described elsewhere [21]. Briefly, 1 × 10^6 ^INS-1 cells, an insulinoma cell line [25], were cotransformed with 1 μg of the plasmids pCDNA-FKBP13linkBla[2] and with 1 μg pCMVSPORT-Bla[1]M182TlinkCENP-A, or with pCMVSPORT-Bla[1]M182TlinkBla[2] alone, or with the positive control plasmids using the FuGENE 6 transfection reagent (Roche Diagnostics, Mannheim, Germany) according to the manufacturer's instructions. After two days, cells were washed with PBS and harvested into 100 μl of 100 mM phosphate buffer, pH 7.0 and lysed by three freeze-thaw cycles. After centrifugation at 13,000 g for 4 min, the supernatant was used to check for β-lactamase activity. In a 96-well microtiter plate each well was loaded with 80 μl of the phosphate buffer, 80 μl dejonized water, 20 μl of phosphate buffer containing 1 mM nitrocefin (EMD Biosciences, San Diego, CA, freshly prepared from a 10 mM stock solution in DMSO) and 20 μl of each supernatant. The absorbance at 496 nm was measured time-dependently in a platereader (Spectra Max 340, Molecular Devices, Sunnyvale, CA). Data after 90 min of incubation were used to calculate the absorbance as percent of control.

## Authors' contributions

The authors contributed equally to this work
